# Seasonal ecophysiology of two páramo species: the dominance of light over water limitations

**DOI:** 10.3389/fpls.2025.1529852

**Published:** 2025-04-14

**Authors:** Adriana Sanchez, Lina M. Mercado, Juan M. Posada, William Kirby Smith

**Affiliations:** ^1^ Departamento de Biología, Facultad de Ciencias Naturales, Universidad del Rosario, Bogotá, Colombia; ^2^ Faculty of Environment, Science, and Economy, University of Exeter, Exeter, United Kingdom; ^3^ UK Centre for Ecology and Hydrology, Wallingford, United Kingdom; ^4^ Department of Biology, Wake Forest University, Winston-Salem, NC, United States

**Keywords:** Chingaza, chlorophyll fluorescence, climate change, photosynthesis, seasonality of carbon uptake, tropical mountains, water potential

## Abstract

Dry and rainy seasons in many ecosystems differ significantly in cloudiness, precipitation, and incident sunlight. These seasonal variations can influence photosynthesis by altering light availability and water stress. This study examines whether light availability or water stress is the primary limiting factor for photosynthesis in páramo plants during the dry and rainy seasons. We measured photosynthetic carbon gain per unit leaf area (*A_n_*), stomatal conductance (*g_s_*), chlorophyll fluorescence (ϕPSII), and leaf water potentials, in two dominant páramo species, *Espeletia grandiflora* and *Chusquea tessellata*, across both seasons. Photosynthetic light-response curves were generated for each species, and statistical analyses assessed the relative influence of environmental factors such as light, temperature, and vapor pressure deficit on An. Contrary to our expectations, An was higher in the dry season despite increased water stress, suggesting that light availability is a stronger driver of carbon assimilation. However, light-response curves showed that *Espeletia grandiflora* exhibited higher potential carbon uptake during the dry season, while *C. tessellata* had greater uptake during the rainy season. Statistical analyses indicated that light was the primary factor influencing *A_n_* in both seasons, though temperature and vapor pressure deficit also played a role for *C. tessellata* in the rainy season. The combination of high solar radiation and elevated leaf temperatures in the dry season facilitated greater carbon assimilation, particularly in *E. grandiflora*. In contrast, the cloudier conditions of the rainy season limited photosynthesis despite reduced water stress. Although *C. tessellata* exhibited high *A_n_* during the dry season, it appeared vulnerable to high radiation and desiccation. These findings emphasize that cloud cover and light availability, rather than water stress alone, are key drivers of páramo plant carbon uptake, with important implications for predicting climate change effects in high-altitude ecosystems.

## 1 Introduction

Seasonal climate changes strongly influence carbon fixation, resource allocation and plant growth. In temperate and boreal regions, seasonality is primarily driven by temperature and day length. In contrast, tropical regions experience low monthly mean air temperature variation and solar radiation is stable year-round (Sarmiento, 1986). However, seasonality plays a crucial role in the tropics, largely dictated by seasonal precipitation changes driven by the northward and southward movement of the Intertropical Convergence Zone (Asmerom et al., 2020; Yuan et al., 2023). Therefore, tropical seasonality differs from that in the temperate and boreal zone, with large seasonal variations in precipitation, cloud occurrence, and incident sunlight (Sanchez et al., 2018; Hughes et al., 2024).

Except for the wettest or driest regions in the Neotropics, heavy rainfall alternates between rainy and dry seasons, either unimodal or bimodal (Sarmiento, 1986). High and dark cloud cover prevails during rainy seasons, leading to reduced incident sunlight, high soil water availability, relatively constant air temperatures (*T*_air_), high relative humidity (RH) and thus, low vapor pressure deficits (VPD) (e.g., Sarmiento, 1986; Smith and Young, 1987; Sanchez et al., 2014, 2018; Hughes et al., 2024). Opposite conditions such as low cloudiness and clear skies, high radiation (or photosynthetic photon flux density; PPFD), low soil water availability, low RH and more extreme air and leaf temperature fluctuations occur during the dry season (Sanchez et al., 2018; Hughes et al., 2024). These conditions can impose water stress and expose plants to low nighttime temperatures, which may limit carbon assimilation.

Seasonal contrasts are more pronounced in extreme environments such as tropical high-altitude ecosystems, located above the tree line at elevations above 3000-3500 m (Sayre et al., 2020). Here, plants endure multiple stressors, including low mean annual temperature (~10°C), frequent frosts (more common in the dry season), high solar radiation, strong winds, as well as temporally variable sunlight regime (Sarmiento, 1986; Rada, 2016; Sanchez et al., 2018). Moreover, stressors vary depending on the season. During the dry season, plant growth may be limited by reduced soil water availability, high VPD, PPFD and *T*_air_, while cold nights can cause photoinhibition by reducing the photochemical efficiency of PSII (Jordan and Smith, 1995). In contrast, insufficient sunlight due to high cloud cover, and suboptimal temperatures for photosynthesis may constrain growth during the rainy season.

The páramo ecosystem of the Northern Andes is of critical importance for the multiple ecosystem services provided (e.g., Buytaert et al., 2011; Benavides et al., 2018; Diazgranados et al., 2021). For example, this ecosystem provides most of the drinking water and hydropower to numerous and highly populated municipalities in Colombia. Biodiversity in this highly diverse ecosystem is also under threat (Madriñán et al., 2013; Peyre et al., 2020; Wheatly et al., 2023). Therefore, understanding how the vegetation responds to the current seasonal variations and stresses is essential for better predicting the impacts of climate change, including rising temperatures and intensified droughts.

Climate models predict that increasing temperatures will displace páramo ecosystems to higher elevations (Buytaert et al., 2010; Feeley et al., 2020; Freeman et al., 2021). These predictions are based on what we know about the response of respiration and photosynthesis to increases in temperature (Berry and Björkman, 1980; Yamori et al., 2013), as well as palynological records of past plant distributions (e.g., Van der Hammen and Hooghiemstra, 2000; Hooghiemstra and van der Hammen, 2004; Morueta-Holme et al., 2015; Flantua and Hooghiemstra, 2018). However, past climate change occurred under different conditions to the ones we have today. Thus, more studies are needed on species and site-specific responses to seasonality.

Here, we examine how temperature, sunlight and precipitation influence carbon assimilation and water stress in a Colombian páramo that provides ca. 70% of the drinking water to the ca. 8 million people capital city, Bogotá (Buytaert et al., 2011). The páramo we studied is characterized by a short three- to four-month dry season and therefore, we hypothesize that water stress during this season would be the primary limiting factor for plant carbon assimilation. Previous studies in Venezuelan páramos have shown that the dry season reduces photosynthesis due to low soil water availability and the concomitant reduction in stomatal conductance (e.g. Rada, 2016). Our aim is to identify the ecophysiological bases driving seasonal differences in photosynthetic carbon assimilation.

## 2 Materials and methods

To understand which climatic conditions may limit carbon assimilation in the páramo of Chingaza National Park, we recorded key ecophysiological *in situ* measurements in two dominant species of the Colombian páramo, over 10 months, during both rainy and dry seasons. These measurements included environmental conditions experienced at the leaf level such as leaf temperature (*T_leaf_*), incident sunlight (*PPFD_l_*), and vapor pressure deficit (*VPD_l_*). We also measured diurnal cycles of chlorophyll fluorescence (ϕPSII) and gas exchange, photosynthetic light response curves, and leaf water potentials (*ψ*) (see below). Measurements were recorded during three to four consecutive days per month to capture daily variations in the abovementioned parameters (N = 30 days, 11 during the rainy season and 19 during the dry season). Individual species leaf-level photosynthetic carbon fixation response to water stress during the dry season, or low incident sunlight during the rainy season were evaluated.

### 2.1 Study area

This study was carried out in the páramo of the Chingaza National Park (4°41′00.2′′N, 73°47′14.5′′W and 3655 m elevation) located ca. 66 km from Bogotá. Chingaza is considered a humid páramo, with the Eastern flank characterized by a unimodal precipitation regime with a rainy season lasting from approximately April through November and a shorter dry season from December to late February or early March (Cleef, 1981; Sarmiento, 1986). Mean annual precipitation between 1987 and 2014 was 2171 mm year^-1^, with years 2011 (2960 mm year^-1^) and 1994 (1843 mm year^-1^) considered as the rainiest and driest years, respectively. Mean monthly precipitation is three times higher during the rainy than during the dry season months (mean dry and rainy season monthly precipitation is 70 mm and 209 mm, respectively; p < 0.001) (Sanchez et al., 2018).

Mean annual air temperature at Chingaza varies between ca. 6 and 10.5°C with maximums close to 25°C and nighttime minimums several degrees below freezing (Cardenas et al., 2002; Sanchez et al., 2018). Diurnal temperature range is larger during the dry season, e.g. daytime maximum air temperature, and nighttime minimum during the rainy season rarely go below freezing (Tol and Cleef, 1994; Cardenas et al., 2002; Sanchez et al., 2018). Saturation water vapor pressure deficit of the air (*VPD*) and incident sunlight (photosynthetic photon flux density, *PPFD*) are substantially higher during the dry season, while windspeed is significantly lower. More details of climate at the Chingaza páramo can be found in Sanchez et al. (2018).

### 2.2 Study species

**Figure 1:**
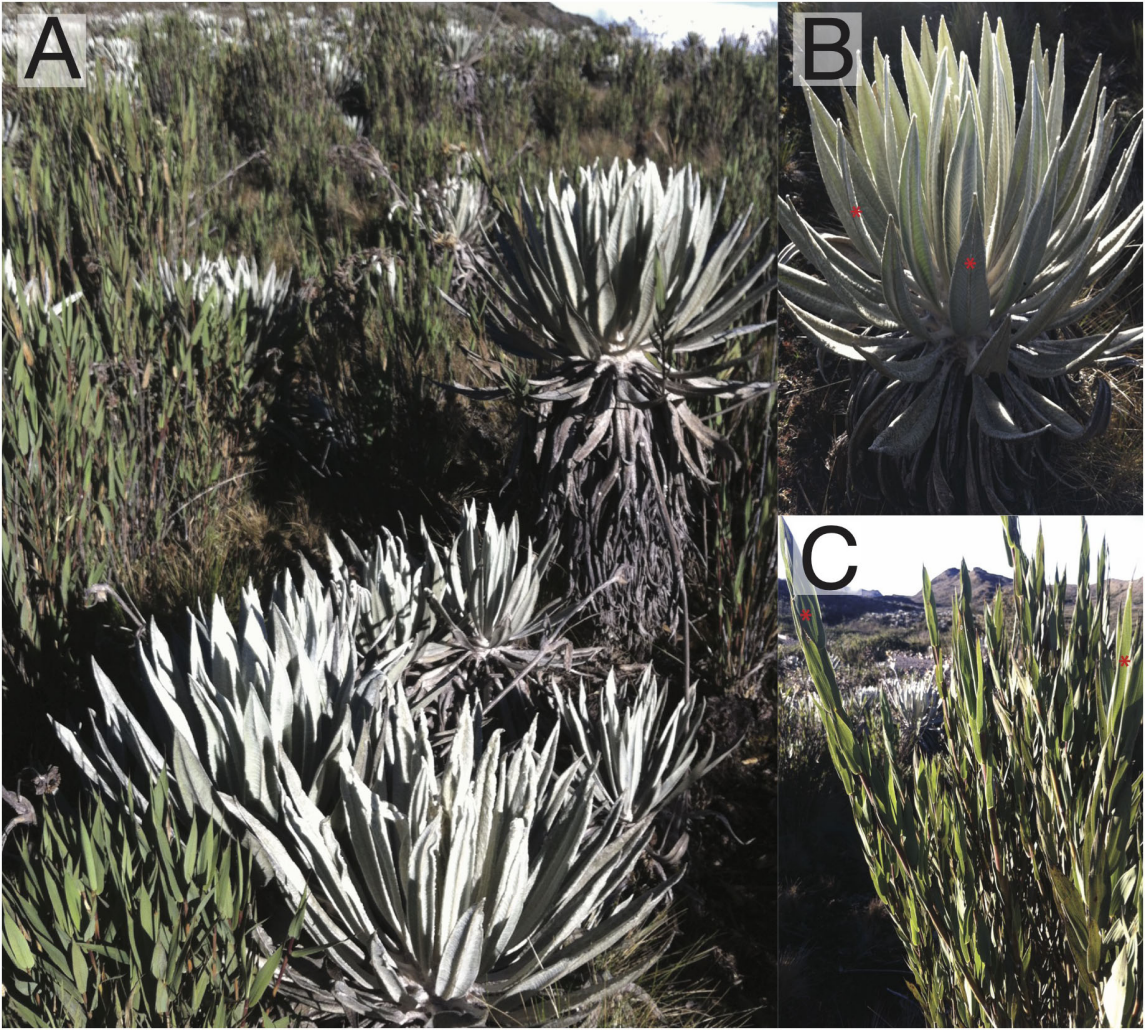
Study area in the Chingaza páramo and the two species studied. **(A)** View of the study area with both species growing next to each other. **(B)** Close-up to *Espeletia grandiflora* (Asteraceae) a caulescent rosette dominant in this páramo. **(C)** *Chusquea tessellata*, a bambu from the family Poaceae common in humid páramos. Red asterisks indicate the model leaves where measurements were recorded.

Two dominant species in the Chingaza páramo were chosen for this study: *Espeletia grandiflora* Bonpl. (Asteraceae) and *Chusquea tessellata* Munro (Poaceae) (**Figure 1**); both species grow sympatrically in the area and represent different functional strategies. *Espeletia grandiflora* is an endemic caulescent rosette of the Eastern Andes of Colombia that grows up to ca. 5 m in height, with leaves covered by a dense, white pubescence. These giant rosettes are characterized for their layer of marcescent leaves around the stem, which protects it from freezing temperatures. Additionally, the central stem contains a pith that stores water, helping the rosettes maintain favorable leaf water potentials even in conditions of limited soil water availability (Goldstein et al., 1984, Estrada et al., 1991; Monasterio and Sarmiento, 1991; Rada, 2016; Rada et al., 2019). *Chusquea tessellata* has a broad distribution from Venezuela to Ecuador between the elevations of ca. 2800 and 4300 m. This plant can grow up to 3 m in height and it is usually found in wet ecosystems (POWO, 2024). Growth rates for *E. grandiflora* are 6-8 cm/year (Fagua and Gonzalez, 2007); for *C. tessellata* growth rates are unknown.

All physiological measurements described below were recorded within 25 m of the weather stations described in Sanchez et al. (2018) during a 10-month period: December 2012 through May 2013, and November 2013 through February 2014.

### 2.3 Environmental conditions at the leaf level

Each month, we selected four healthy-appearing individuals per species and chose one fully mature, undamaged leaf per plant from the same approximate stem position to ensure similar age between individuals. We changed individual plants every month but consistently chose mature, healthy-appearing, fully expanded and south-facing leaves. A total of 40 leaves per species were monitored during the 10-month interval. For each individual leaf we continuously measured temperature (*T_leaf_*) and incident sunlight (*PPFD_l_*), using fine-wire (36 ASU gauge), copper-constantan thermocouples and GaAsP light sensors (G1118 Hamamatsu) respectively, as described in more detail in Sanchez et al. (2018). Values for the vapor pressure deficit at the leaf level (*VPD_l_*) were obtained from each gas exchange measurement (see below) based on *T_leaf_*, saturation vapor pressure, and vapor pressure in the sample chamber (Equations 14-24 and 14-21, LiCor LI-6400XT instruction manual).

### 2.4 Diurnal cycles of photosynthetic gas exchange and chlorophyll fluorescence

On all sample days, we measured leaf net photosynthesis to characterize the daily cycle and seasonal differences, at approximately 2 h intervals between 0700 and 1500 h, and at 1600 h using a LiCor LI-6400XT Portable Photosynthesis System (LiCor, Lincoln, NE) equipped with a clear chamber. Measurements were made on leaves of marked individuals (N = 4 for each species per month) in their natural orientations and in leaves with the *T_leaf_* and *PPFD_l_* sensors attached. Therefore, a total of six spot measurements we recorded per day, per leaf. Air temperature inside the measurement chamber was kept at ambient values and reference CO_2_ was maintained at 400 ppm. Relative humidity was also held at or near ambient values (<10% deviation), but downward adjustments of RH were more frequent during the rainy season due to high humidity alerts in the gas exchange system. In some cases, *C. tessellata* leaves did not cover the entire area of the chamber. In those cases, leaves were marked in the places where gas exchange was recorded and at the end of the sampling period were harvested and taken to the laboratory for leaf area determination. One sided leaf area was measured using a high resolution scanner (HP Scanjet G4050) and the software ImageJ (Schneider et al., 2012). We then used the measured leaf areas within the gas exchange calculations.

Measurements of chlorophyll *a* fluorescence were done to determine the chlorophyll fluorescence or maximum quantum yield of PSII (ϕPSII) in dark-adapted (predawn only) and light-adapted (all daytime measurements) states. PSII is one of the most sensitive parts of the photosynthetic apparatus, and as the capacity to process light decreases, fluorescence is one of the mechanisms to decrease unused energy (Braun et al., 2002). ϕPSII is measured through the *F_v_/F_m_* ratio, used to evaluate the efficiency of photosynthesis and serves as proxy to photosynthetic activity (Krause et al., 2010). Reduced values of ϕPSII indicate stress, photosynthesis downregulation and photoinhibition (Bartold and Kluczek, 2024), which can translate into a decline in net carbon assimilation (Dujardyn and Foyer, 1990). Fluorescence measurements can thus provide key information on stress related to seasonality and the diurnal cycle. These measurements were made on the same days as gas exchange using a PAM fluorometer (model FMS-2, Hansatech Instruments, Norfolk, UK), emitting an amber (594 nm) saturating pulse of 2 s long, 3 mmol m^-2^ s^-1^, at approximately 0530 (predawn), and every two hours through 1700 h. For the light-adapted measurements, the quantum yield efficiency of PSII in the light (or *F’_v_*/*F’_m_*) was measured by placing the measurement clip in an open position and recording *F’_v_*/*F’_m_* (or [*F’_m_* – *F’_O_*]/*F’_m_*) immediately after a 1–2 second dark period, following the protocol described in Hughes et al. (2014).

For all statistical analyses, we first tested data using the Shapiro–Wilk test (normality determined as p > 0.05) and Levene test. Because normality criteria were not met after transformations, means of the dry and the rainy season were compared using the Mann-Whitney test. Mean net photosynthesis (*A_n_*), intercellular CO_2_ concentration (*Ci*), stomatal conductance (*g_s_*), transpiration (*E*) and water use efficiency (WUE = *A_n_/E*), as well as ϕPSII for the dry and the rainy season data were analyzed separately for *E. grandiflora* and *C. tessellata*. Rain events occurred during both seasons but were most frequent during the rainy season; when leaves were visibly wet from rainfall, those measurements were excluded from the analyses of *g_s_, E*, and *WUE.* All tests were accomplished using R version 4.1.2 (R Core Team, 2021). Statistical significance was determined at p < 0.05.

### 2.5 Photosynthetic light response

To determine the influence of *PPFD* on *A*, light response curves were measured for each species using the LiCor LI-6400XT equipped with a red/blue light source (6400-02B LED, LiCor). Measurements were taken between 0900 and 1100 h on the same leaves for which gas exchange measurements were taken. Experimental PPFD values used were: 2000, 1500,1000, 750, 500, 300, 200, 100, 50, 25, 10, and 0 μmol m^-2^ s^-1^. Measurements began at the maximum PPFD and decreased stepwise allowing 2-3 min acclimation between measurements. Photosynthetic quantum use efficiency (quantum yield, in μmol m^-2^ s^-1^ CO_2_/μmol m^-2^ s^-1^ PPFD) was calculated as the slope of the best-fit regression line, for the linear portion between 0 and 300 μmol m^-2^ s^-1^ PPFD. To determine whether each species light response of photosynthesis differed between the dry and rainy season, regressions for all PPFD values were compared using one-way ANCOVA, with light intensity and season used as continuous co-variables. Statistical analyses were applied using R and p < 0.05. For the dry season, four individuals per species and per month were measured (during six months), accounting for a total of 24 light response curves. Given that the environmental conditions during the rainy season were particularly difficult to obtain reliable gas exchange measurements, we could only measure four individuals per species during April of 2013.

The photosynthetic light response curves were obtained by fitting a non-rectangular hyperbola given by the following equation (Thornley, 1976):


(1) $$A_{n}=\;\frac{\phi I+A_{sat,g}-\;\sqrt{{\left(\phi I+A_{sat,g}\right)}^{2}-4\theta \phi IA_{sat,g}}\;}{2\theta }-\;R_{d}$$

where *A_n_* is net CO_2_ assimilation, *I* is instantaneous *PPFD* incident on the leaf, *f* is the apparent quantum yield, *A_sat,g_* is the light saturated rate of gross assimilation, *θ* is the curvature, and *R*_d_ is foliar dark respiration (Equation 1). These four parameters were estimated with R using a numerical optimization method based on a Nelder–Mead, quasi-Newton and conjugate-gradient algorithm. The specific method used was “L-BFGS-B” (Byrd et al., 1995), which is programmed in the package *optim*.

### 2.6 Leaf water potential

Under a marked precipitation seasonality and high evaporative demand during the dry season, plants in Chingaza páramo are under a limited water availability during three to four months per year (~December to March). Leaf water potential (*ψ*) measures the potential energy of water relative to pure water and is an important metric of plant water status. Water stress may cause plants to close their stomata and this closure may cause reductions in net photosynthetic rates, which affects plant growth and survival (Rada et al., 2012, 2019). To capture daily and seasonal differences in plant water status, we measured *ψ* on the same days as photosynthetic gas exchange measurements, in the morning between 0600 and 0700 h, at midday between 1200 and 1300, and in the late afternoon between 1630 and 1730 for both *E. grandiflora* and *C. tessellata* (N = 3 per month per species). South-facing leaves used for *ψ* measurements were chosen randomly from the field site, close to the same individual plants undergoing gas exchange measurements. For each *ψ* measurement, a leaf was excised from the plant using a razor blade and immediately (within <1 min) placed in a Scholander-Hammel pressure chamber (1000 model, PMS Instruments, Corvallis, OR) to estimate *ψ*. Data were tested for normality and when data could not be transformed to achieve normality, means of the dry and the rainy season were compared using the Mann-Whitney test in R.

### 2.7 Relationships between environmental and gas exchanges variables

Using the diurnal cycles of *A_n_*, we also evaluated the variation of leaf *A_n_* with leaf temperature (*T_leaf_*), vapor pressure deficit (*VPD_l_*) and incident sunlight (*PPFD_l_*). Additional relationships between *A_n_* and other gas exchange variables such as *C_i_* (an indication of the balance between liquid phase and gas phase conductances to CO_2_), *g_s_* and *E*, as well as ϕPSII and *ψ* were also examined. We also tested the relationship between *VPD_l_* and *g_s_.* The association between *A_n_* and *ψ* was evaluated by approximating *ψ* at the next measurement time of *A_n_*, that is 0600 h to 0700 h, 1200 to 1300 h and 1700 to 1600 h. The statistical strength of these relationships was evaluated using the Spearman rank correlation analysis.

Additionally, a single multiple linear regression model was used to evaluate how all the abiotic factors (*T_leaf_*, *PPFD_l_* and *VPD_l_*) relate with *A_n_*, for each species and season. For the rainy season, the data were log-transformed for both species to meet the assumptions of linear models.

## 3 Results

### 3.1 Seasonal patterns in gas exchange

**Figure 2:**
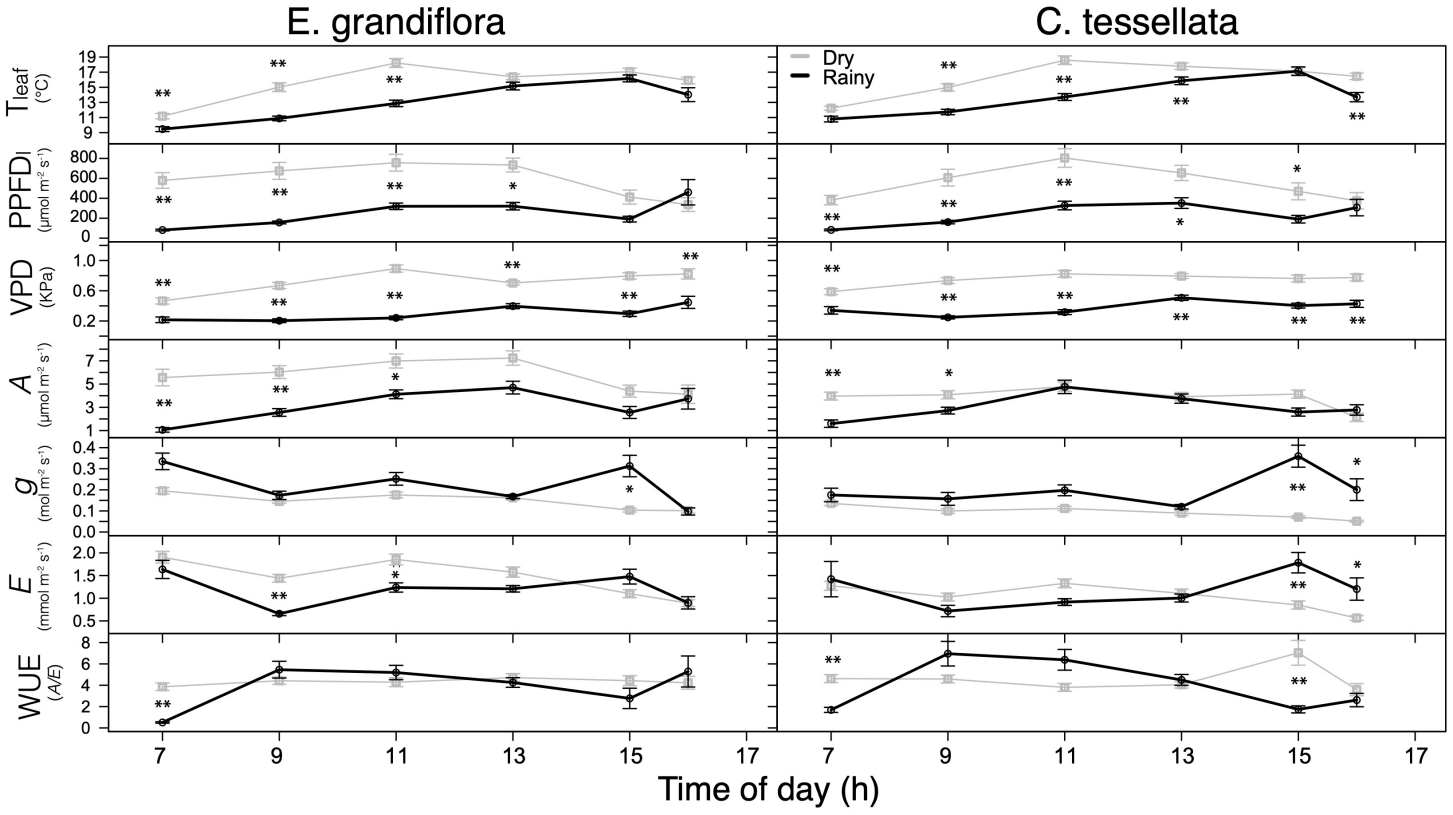
Measured leaf temperature (T_leaf_), photosynthetic photon flux density at the leaf (PPFD_l_), vapor pressure deficit at the leaf (VPD_l_), photosynthetic carbon gain per unit leaf area (*A_n_*), water vapor stomatal conductance (*g_s_*), evapotranspiration (*E*) and instantaneous water use efficiency (WUE = *A_n_/E*) for the dry (grey) and rainy (black) seasons in *E. grandiflora* and *C. tessellata*. Values for time intervals correspond to means of 3-4 days per month ± S.E.; the dry season comprises six measurement months while the rainy season, four. Significant differences are indicated: *p < 0.05; **p ≤ 0.01.

Daytime leaf temperatures (*T_leaf_*) were significantly higher during the dry than during the rainy season in both species (W = 27961, p < 0.001; 16.3 versus 13.8°C in *C. tessellata* and W = 33338, p < 0.001; 15.6 versus 13.0°C in *E. grandiflora*) with maximum values occurring near noon (**Figure 2**). Leaf level incident sunlight (*PPFD_l_*) was also significantly higher during the dry than during the rainy season in both species (p < 0.001; **Figure 2**) (W = 27315; 569 and 252 μmol m^-2^ s^-1^ in *C. tessellata* and W = 32622; 613 and 257 μmol m^-2^ s^-1^ in *E. grandiflora*).

*A_n_* was highest during the dry season for both studied species (**Figure 2**). On average, *A_n_* for *E. grandiflora* was 6.0 and 3.4 μmol m^-2^ s^-1^ (W = 31051, p < 0.001), while *A_n_* for *C. tessellata* was 3.9 and 3.3 μmol m^-2^ s^-1^ (W = 22719, p < 0.05) during the dry and the rainy season, respectively. Despite increased water stress in the dry season, carbon assimilation (*A_n_*) was significantly higher in both species. Maximum *A_n_* values in *E. grandiflora* occurred at 1300 h in both the dry (24.1 μmol m^-2^ s^-1^) and rainy season (12.1 μmol m^-2^ s^-1^), while in *C. tessellata*, these maxima also occurred at 13 h in both seasons but rainy season values where higher (13.8 and 15.5 μmol m^-2^ s^-1^, respectively).

Water vapor conductance from the leaf, a close estimate of stomatal conductance to water vapor (*g_s_*), was significantly higher during the rainy (0.16 and 0.20 mol m^-2^ s^-1^ for *C. tessellata* and *E. grandiflora*, respectively) than during the dry season (0.09 and 0.15 mol m^-2^ s^-1^ for *C. tessellata* and *E. grandiflora*, respectively) (W = 6765, W = 7217 for *C. tessellata* and *E. grandiflora*, respectively; p < 0.001; **Figure 2**). **Figure 2** also shows that mean transpiration (*E*) had a pattern similar to *g_s_* in both species, with an increase at 15 h. However, *E* was not significantly different (p > 0.05) between seasons in *C. tessellata* (W = 10352; 1.05 vs. 1.12 mmol m^-2^ s^-1^ in the dry and rainy season, respectively). In contrast, in *E. grandiflora* there were significant differences in *E* between seasons (W = 11173, p < 0.001), with higher values (1.47 mmol m^-2^ s^-1^) in the dry season compared to the rainy season (1.16 mmol m^-2^ s^-1^) (**Figure 2**). There were no significant differences (p > 0.05) in water use efficiency (*WUE*) between the dry and rainy season in either *E. grandiflora* or *C. tessellata* (W = 9882 and W = 11798; **Supplementary Figure 1**). *WUE* was more strongly related to *A_n_* and in some cases, there was no significant relationship between *WUE* and *E* (dry season for *E. grandiflora;* **Supplementary Figure 1**). During the rainy season the intercellular CO_2_ concentration (*C_i_*), was significantly higher (p < 0.01; results not shown). Mean *C_i_* for *E. grandiflora* was 309 and 339 ppm in the dry and rainy season (W = 6330), respectively, and in *C. tessellata* 303 and 344 ppm (W = 5869).

**Figure 3:**
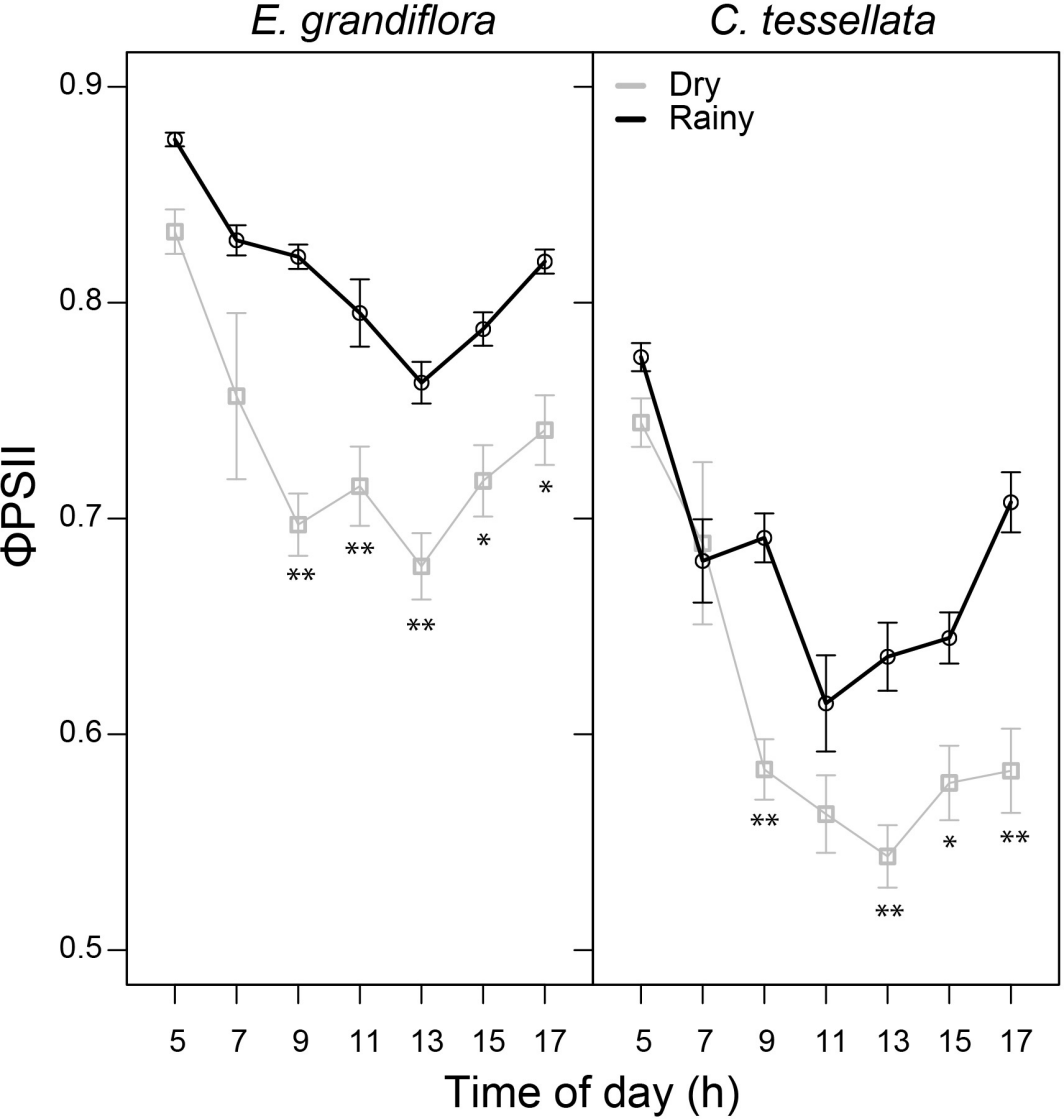
Quantum use efficiency of the photosystem II (θPSII) for *E. grandiflora* and *C. tessellata* during the dry and rainy seasons. Values from 7 to 17 h correspond to light-adapted F_v_/F_m_ measurements. Values for time intervals correspond to means of 3-4 days per month ± S.E.; the dry season comprises six measurement months while the rainy season, four. Significant differences are indicated as * (p < 0.05) and ** (p ≤ 0.01).

**Figure 3** shows that predawn ϕPSII was similar in the dry and the rainy season for both species, 0.88 and 0.83 for *E. grandiflora* (W = 35881) and 0.77 and 0.74 for *C. tessellata* (W = 39331), respectively. The dry season had significantly lower average values of daytime ϕPSII in both species (p < 0.001), although, *E. grandiflora* had higher values (0.72) than *C. tessellata* (0.60). In both species there was a marked decrease in ϕPSII at 7 and 9 h, and a recovery after 13 h. The absolute minimum and lowest mean of ϕPSII was 0.24 and 0.54 in *C. tessellata* and occurred at 15 h. In *E. grandiflora* these values were 0.31 and 0.67 at 13 h (**Figure 3**).

### 3.2 Seasonality in photosynthetic light response

**Figure 4:**
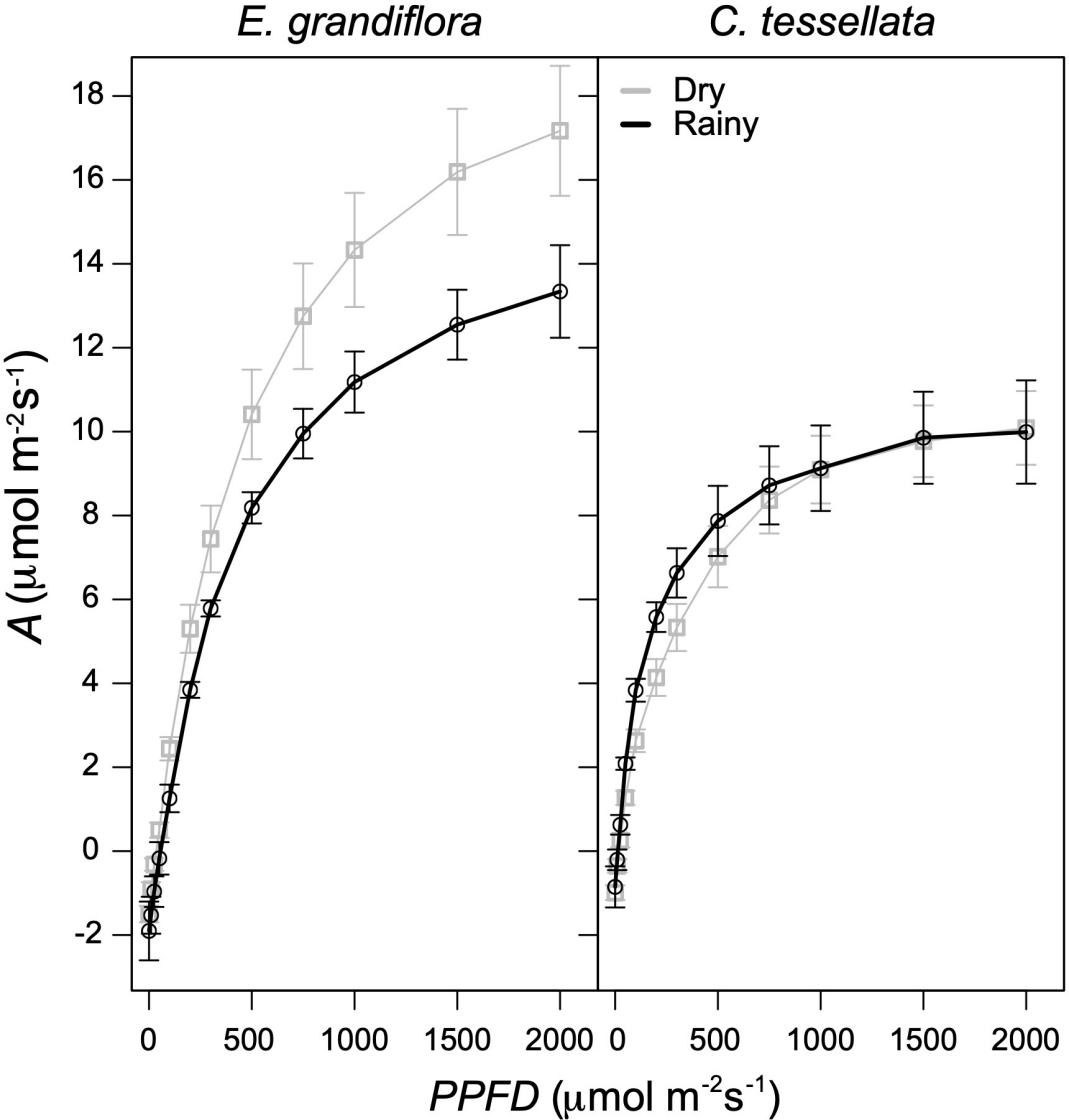
Light response of photosynthesis (*A*) for *E. grandiflora* and *C. tessellata*. All curves fit a non-rectangular hyperbola (see **Equation 1**). Dry season curves correspond to 24 curves while rainy season to four.

ANCOVA analysis of photosynthetic light-responses showed that *A* was significantly related to *PPFD* in both species (F = 396 and 537.3 for *C. tessellata* and *E. grandiflora*, respectively*;* p < 0.001), but there was no significant difference between seasons (F = 2.56 and 2.84 for *C. tessellata* and *E. grandiflora*, respectively*;* p > 0.05; **Figure 4**). These results indicate that if *PPFD* is not limiting, photosynthesis (*A*) does not differ between seasons. *Espeletia grandiflora* had higher *A* values than *C. tessellata* with an absolute maximum *A* at 2000 μmol m^-2^ s^-1^ (*A_sat_*) of 34.6 μmol m^-2^ s^-1^ in the dry season and 15.3 μmol m^-2^ s^-1^ in the rainy season (results not shown). Mean *A_sat_* (at 2000 μmol m^-2^ s^-1^) during the dry season was 17.2 μmol m^-2^ s^-1^ and 13.3 μmol m^-2^ s^-1^ during the rainy season for *E. grandiflora* (**Figure 4**). Highest *A_sat_* values in *C. tessellata* occurred during the dry season (16.2 μmol m^-2^ s^-1^ compared to 11.9 μmol m^-2^ s^-1^ during the rainy season; results not shown). However, mean *A_sat_* was similar in both seasons (10.1 μmol m^-2^ s^-1^ in the dry and 10.0 in the rainy season; **Figure 4**). **Figure 4** also shows that leaf dark respiration values obtained from the light response curves were higher in *E. grandiflora*, compared to *C. tessellata*, with the absolute respiration of 3.0 μmol m^-2^ s^-1^ during the dry season and 2.2 μmol m^-2^ s^-1^ in the rainy season (results not shown), while mean respiration in the dry season was 1.5 and 1.6 μmol m^-2^ s^-1^ in the rainy season (**Figure 4**). In *C. tessellata* the lowest respiration values from the light response curves during the rainy season were 2.5 compared to 2.0 μmol m^-2^ s^-1^ in the dry season (results not shown); mean values were 0.9 and 1.0 μmol m^-2^ s^-1^ in the rainy and dry seasons, respectively (**Figure 4**). Quantum use efficiency was higher in *E. grandiflora* compared to *C. tessellata* and higher in the dry versus the rainy season (0.031 vs. 0.026 μmol m^-2^ s^-1^). In contrast, *C. tessellata* had higher efficiency during the rainy season (0.025 vs. 0.020 μmol m^-2^ s^-1^ for the dry season).

### 3.3 Seasonality in leaf water potential (ψ)

**Figure 5:**
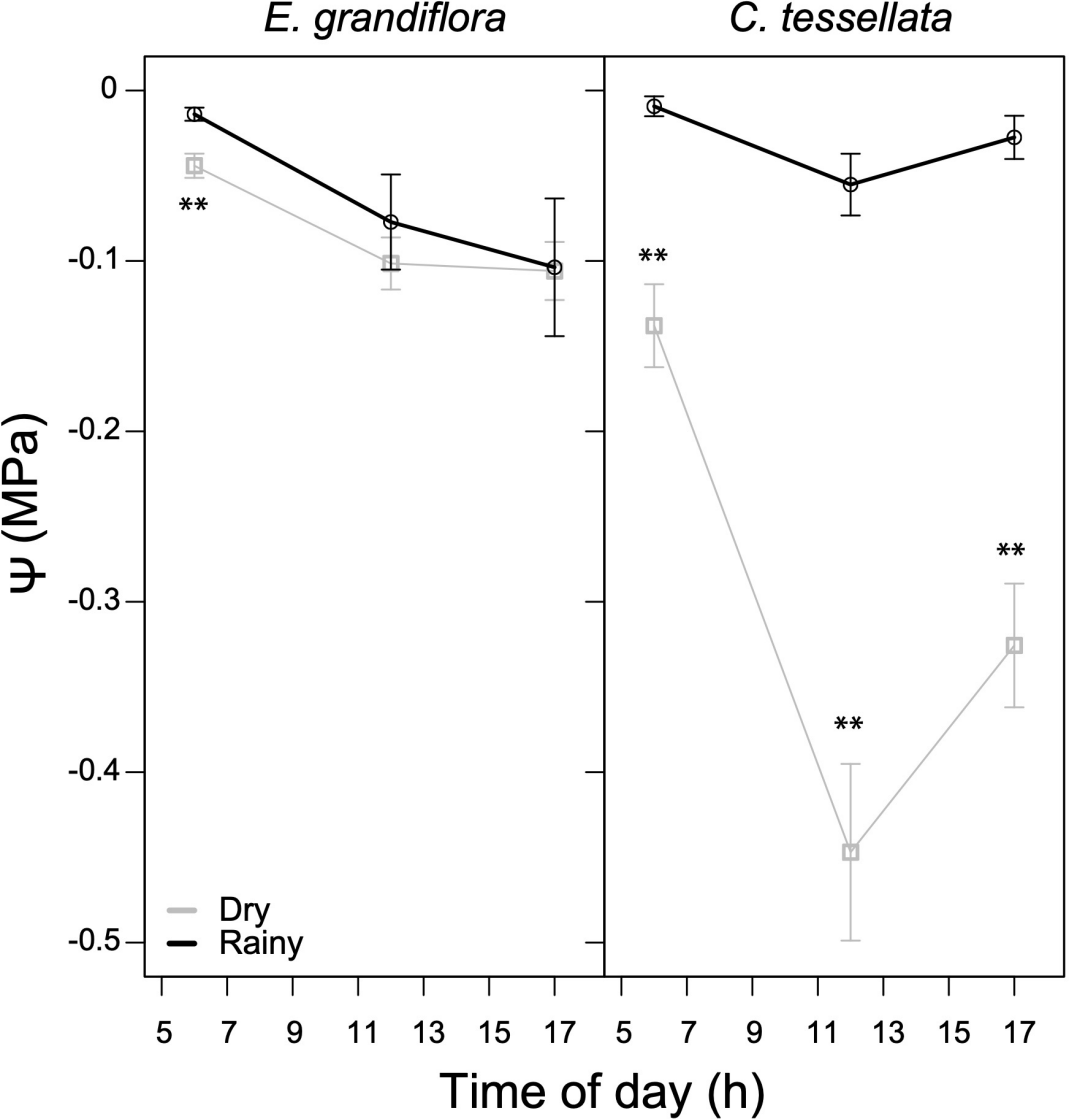
Diurnal leaf water potentials (*ψ*) for the dry and rainy seasons in *E. grandiflora* and *C. tessellata*. Values for time intervals correspond to means of 3-4 days per month ± S.E.; the dry season comprises six measurement months while the rainy season, four. Significant differences are indicated as **(p ≤ 0.01).

There was a significant difference in average daily *ψ* between seasons in *C. tessellata* (W = 1187, p < 0.001), with the dry season generating dryer (more negative) values. During the rainy season, mean *ψ* was -0.03 MPa, while it declined substantially to -0.3 during the dry season. *Espeletia grandiflora* had much more similar *ψ* values during both seasons, with a mean *ψ* of -0.06 for the rainy and -0.08 MPa for the dry season (W = 2862.5, p < 0.05). In *C. tessellata*, the lowest *ψ* values were at noon with markedly differences in minima between seasons (-0.43 MPa, dry season and -0.05 MPa, rainy season), while *E. grandiflora* had the lowest *ψ* (-0.10 MPa) at 17 h during both seasons. Overall, in **Figure 5** shows that *E. grandiflora* had higher *ψ* values (less negative) than *C. tessellata*, especially during the dry season. These results suggest that *C. tessellata* is more sensitive to drought.

### 3.4 Environmental and gas exchange relationships with A_n_

Photosynthesis (*A_n_*) (**Figure 2**) was significantly and positively correlated with *PPFD_l_*, *T_leaf_* and *VPD_l_* (p < 0.01) in both species and seasons (**Supplementary Table 1**). However, this correlation was consistently stronger with incident sunlight (*PPFD_l_*), especially during the rainy season (r_s_ = 0.90 and 0.81 in *E. grandiflora* and *C. tessellata* respectively). Although *T_leaf_* and *A_n_* were correlated, this relationship was, overall, not very strong during the dry season (r_s_ = 0.34 and 0.18 in *E. grandiflora* and *C. tessellata* respectively). The correlation between *VPD_l_* and *A_n_* was positive and significant in both species and seasons, but it was stronger during the rainy in *E. grandiflora* compared to the dry season (r_s_ = 0.40 vs. 0.31). For *C. tessellata* the correlation was the same (r_s_ = 0.19) in both seasons (**Supplementary Table 1**). The linear model showed that *PPFD_l_* was the only significant factor on *A_n_* for *C. tessellata* and *E. grandiflora* during the dry season (r^2^ = 0.1 and r^2^ = 0.5, respectively; p < 0.0001) and for *E. grandiflora* during the rainy season (r^2^ = 0.7; p < 0.0001). During the rainy season, *PPFD_l_* and *VPD_l_* had a significant effect on *A_n_* for *C. tessellata* (r^2^ = 0.6; p < 0.0001).

All correlations between *C_i_*: *A_n_*, and *g_s_*: *A_n_* were mostly negative and significant (p < 0.0001), except for *g_s_*: *A_n_* in *C. tessellata* during the rainy season (**Supplementary Table 1**). Interestingly, the relationship between *A_n_* and *g_s_* was positively correlated during the dry season, but negatively correlated during the rainy season (**Supplementary Figure 2**). Other correlations such as *A_n_* versus ϕPSII and *A_n_* versus *ψ* were significant and negatively correlated in *E. grandiflora* during both seasons and in the rainy season for *C. tessellata* (**Supplementary Table 1**). These were not significant (p > 0.05) in *C. tessellata* during the dry season.

## 4 Discussion

We hypothesized that reduced precipitation during the dry season would decrease carbon assimilation in *E. grandiflora* and *C. tessellata*. However, both species exhibited higher assimilation in the dry season, suggesting that incident sunlight has a stronger influence on photosynthesis than water availability in this páramo ecosystem (Sanchez et al., 2018). Studies in the Venezuelan páramo have linked reduced precipitation to lower carbon assimilation (e.g., Rada, 2016; Rada et al., 2019), but our results in Chingaza show that sunlight is the primary limiting factor (**Figures 2**, **4**). As shown in Sanchez et al. (2018), the dry season is characterized by higher solar radiation and air temperatures. These conditions resulted in higher carbon assimilation, especially in *E. grandiflora*, despite drier conditions (**Figures 2**, **5**). However, our results also suggest that *C. tessellata* appears more vulnerable to desiccation and intense radiation during the dry season (**Figures 3**, **5**).

### 4.1 Species level response to seasons

The species used in this study correspond to two very different plant families: Poaceae (*C. tessellata*) and Asteraceae (*E. grandiflora*). Both plant families are representative and dominant in the páramo ecosystem. However, *C. tessellata* is only present in humid páramos (POWO, 2024), differing from other Poaceae such as *Calamagrostis* and *Festuca* that are constitutive of the ecosystem (Peyre et al., 2015). However, *E. grandiflora* and *C. tessellata* responded similarly in that both species had higher *T_leaf_*, *A_n_* and *E*, along with lower *g_s_* and ϕPSII during the dry season, compared to the rainy season (**Figure 2**). Even though the weather conditions during the dry season are more extreme (Sanchez et al., 2018), both species fixed more carbon during this season. Therefore, under the ca. nine months of rainy season typical of Chingaza, there may be a stronger limitation to *A* from cloud cover and lower incident sunlight (*PPFD*) than either temperature or precipitation.

We expected our results to agree with findings from other studies from the páramo in Venezuela. However, Venezuelan páramos receive less precipitation, in general, compared to the Colombian páramos (Llambí and Rada, 2019; Rada et al., 2019). Additionally, the dry season in Venezuelan páramos lasts longer than in Chingaza (four vs. three months), and it is the main limiting factor for plant growth, reproduction and survival (Monasterio, 1980). The more extreme dry season conditions in Venezuela could result in higher water conservation (i.e., stomatal closure) at the expense of carbon assimilation. Therefore, different life forms in Venezuela (e.g., giant rosettes, forbs, shrubs and trees) consistently show higher carbon assimilation during the rainy season (e.g., Rada et al., 1996; Rada et al., 1998, 2012, 2019).

Despite the more extreme conditions of the Venezuela páramos, reported average *A_n_* values there exceed those reported in this study. For instance, giant rosettes of *Espeletia* have average *A_n_* of 6 μmol m^-2^ s^-1^ (Rada, 2016; Rada and Navarro, 2022), compared to 5.3 μmol m^-2^ s^-1^ in *E. grandiflora* in Chingaza. *Chusquea* species in Venezuelan páramos registered *A_n_* values between 5–8 μmol m^-2^ s^-1^ (Ely et al., 2019), while *C. tessellata* in this study averaged 3.75 μmol m^-2^ s^-1^. Interestingly, light response curves (LRCs) in *C. tessellata* remained stable across seasons, saturating at ca. 1500 μmol m^-2^ s^-1^, while *E. grandiflora* showed seasonal differences (**Figure 4**). This suggests that in *E. grandiflora*, light (PPFD) may limit carbon assimilation more than in *C. tessellata*, contrasting with previous findings in the Colombian páramo where light was not considered limiting (different species from this study; García-León, 2015).

Stomatal conductance (*g_s_*) in Venezuelan páramos is generally low for giant rosettes and other dominant species (Goldstein et al., 1984, 1989; Rada et al., 1998, 2012; Sandoval et al., 2019). During the dry season, mean *g_s_* for five species at 3600–4200 m.a.s.l was 0.05 mol m^-2^ s^-1^ (Rada et al., 2019), while in this study, *E. grandiflora* at 3600 m was three times higher (0.15 mol m^-2^ s^-1^). For the rainy season, *E. grandiflora* maintained higher *g_s_* values (0.20 mol m^-2^ s^-1^) compared to Venezuelan giant rosettes (0.11 mol m^-2^ s^-1^). This likely reflects higher water availability in Chingaza (ca. 2171 mm annual precipitation; Sanchez et al., 2018) compared to Venezuelan páramos (760-1360 mm; Llambí and Rada, 2019; Rada et al., 2019). Higher *g_s_* suggests that *E. grandiflora* can maintain higher stomatal opening, potentially allowing carbon assimilation under less water-limited conditions.

Higher chlorophyll fluorescence (ϕPSII) in the rainy season indicates less light stress compared to the dry season, a pattern also observed in high altitude ecosystems during cloud immersion (Sanchez et al., 2014; Hughes et al., 2015; Leon-Garcia and Lasso, 2019). This decline in ϕPSII was significantly correlated with lower *A_net_* (except for *C. tessellata* during the dry season), suggesting that drier conditions (IPCC, 2021) and/or less cloud cover (Hughes et al., 2024) could intensify photoinhibition, potentially limiting net carbon assimilation.

Giant rosettes in Chingaza maintained higher leaf water potentials (*ψ*) compared to Venezuelan páramos, with values in *E. grandiflora* above -0.15 MPa at peak dry seasons (**Figure 5**). In contrast, values reported in Venezuela were -0.5 to -1.0 MPa (Rada, 2016; Rada et al., 2019). This is likely due to adaptations such as stomatal regulation and water storage in the stem pith (e.g., Monasterio, 1986; Meinzer et al., 1994; Azócar and Rada, 2006; Rada, 2016), making them more resilient to changes in water availability. The bamboo *C. tessellata* reached minimum ψ values between -1.5 and -1.8 MPa during the dry season, similar to other *Chusquea* in Venezuela (Ely et al., 2019), suggesting more vulnerability to dry-season water stress. However, under current conditions, this does not significantly affect carbon assimilation (**Figure 2**).

Despite stronger seasonal drought in Venezuelan páramos, *A_n_* values were higher than in Chingaza. This difference is likely driven by differences in sunlight availability, as Chingaza experiences persistent cloud cover, reducing incident sunlight (Sanchez et al., 2018; Hughes et al., 2024). Less sunlight may limit photosynthetic rates more than water availability in Chingaza páramo. Additionally, the species studied in Chingaza respond differently to seasonality, with *C. tessellata —*restricted to humid páramos— being particularly vulnerable. As climate change intensifies dry seasons and seasonal variability (Cresso et al., 2020), these sensitive species could serve as indicators of drought and light stress, offering insight into future impacts on carbon assimilation.

### 4.2 The importance of understanding seasonal response patterns

The seasons in the tropical high-altitude páramo provide insight into how varying weather conditions throughout the year influence carbon assimilation. Therefore, studying the ecophysiology of representative species under these contrasting seasons can provide key data for making more accurate predictions of the possible consequences of future climatic change. In the Northern Andes of Colombia, these changes include an increase in temperature (Buytaert et al., 2011; Pabón-Caicedo et al., 2020), wide uncertainties regarding the precipitation, with a -17 to +15% changes (Ruiz-Carrascal, 2023) and a higher frequency and intensity of extreme events such El Niño Southern Oscillation (ENSO) (Pabón-Caicedo et al., 2020; IPCC, 2021).

The ecophysiological effects of changes in temperature and precipitation characteristic of the temperate and boreal regions have received, by far, most of the attention in the literature (e.g., Cunningham and Read, 2002; Feeley et al., 2017), while tropical ecosystems remain poorly studied. Moreover, differences in sunlight regimes and concomitant cloud cover have received even less attention in the literature (Hughes et al., 2024), despite being a central factor for the carbon budget of plants. Our findings suggest that light availability, rather than water stress, plays a dominant role in páramo carbon assimilation in Chingaza (Colombia). This could also be the case for other humid páramos in Colombia or other high altitude tropical ecosystems, or even elsewhere where future climate change scenarios project more precipitation, which also means cloudier conditions (e.g., Pabón-Caicedo et al., 2020; Hughes et al., 2024; Rubiano et al., 2025). Future conservation efforts should focus on understanding how shifting cloud regimes, rather than precipitation alone, will shape plant responses in these ecosystems.

If the dry seasons become drier or if the frequency and intensity ENSO increases (e.g., Cresso et al., 2020), species such as the bamboo *C. tessellata* may be more at risk. More precipitation and thus, more cloud cover could, on the other hand, impose carbon assimilation limitations on the giant rosette *E. grandiflora*. Given that most páramo plants can withstand broad temperature changes between ca. -5 C to 25°C in a single day (Sanchez et al., 2018; Rada et al., 2019), temperature alone does not seem as the most important abiotic factor determining the distribution and survival of these plants. Additionally, evidence from Venezuela has shown that several species are operating below their optimal photosynthetic temperature (Rada et al., 1992; Cabrera et al., 1998; Rada, 2016).

Changes in the ecosystem’s vegetation associated with the susceptibility of individual species to future climate change will likely result in new mixed communities (Young et al., 2011; Lurgi et al., 2012; Mavárez et al., 2019; Peyre et al., 2020; Caballero-Villalobos et al., 2021; Duchicela et al., 2021; Peyre, 2022). In these communities we would expect to have plants from the upper Andean forest coexisting with páramo species, as well as an increase in invasive species. This has the potential to impact biodiversity conservation, and important services such as carbon storage and water supply for cities, agriculture and hydropower (Buytaert et al., 2011). However, to understand these potential impacts, it is necessary to conduct studies in other locations and including more species. Additionally, measuring cloud cover to understand the impacts of radiation (Hughes et al., 2024) as well as experiments addressing the impacts of drought are necessary. Although páramo plants have been shown to have high heat tolerance (e.g., Leon-Garcia and Lasso, 2019), more information is needed on their response to increasing temperatures as well as the response to CO_2_ (e.g., *A*-*C_i_* curves). Therefore, more *in situ* experiments will provide valuable information about the possible response of the páramo ecosystem to climate change.

## Data Availability

The datasets presented in this study can be found in online repositories. The names of the repository/repositories and accession number(s) can be found below: https://doi.org/10.5281/zenodo.13713539; https://doi.org/10.5281/zenodo.13306782.
