# Supplementary material for: Seasonal ecophysiology of two páramo species: the dominance of light over water limitations
Source: Front Plant Sci. 2025 Apr 14;16:1529852. doi: 10.3389/fpls.2025.1529852 (PMC12034633; doi:10.3389/fpls.2025.1529852)
Supplement: Supplementary file 1 [file DataSheet1.docx]

**Supplementary Table 1**. Spearman rank correlation test results between net photosynthesis (*A_n_*) and leaf temperature (*T_leaf_*), vapor pressure deficit (*VPD_l_*) and incident sunlight (*PPFD_l_*), as well as *C_i_* (intercellular CO_2_ concentration), stomatal conductance (*g_s_*), transpiration (*E*), chlorophyll fluorescence or maximum quantum yield of PSII (ϕPSII) and leaf water potential (*ψ*). The test was performed for *Chusquea tessellata* and *Espeletia grandiflora* during the dry and rainy season.

| **Species** | **Season** | **Correlation** | **Spearman rho (r_s_)** | **p-value** |
| --- | --- | --- | --- | --- |
| *C. tessellata* | dry | *A_n_* - *T_leaf_* | 0.18 | 0.002 |
|  |  | *A_n_* - *VPD_l_* | 0.19 | < 0.001 |
|  |  | *A_n_* - *PPFD_l_* | 0.50 | < 0.0001 |
|  |  | *A_n_* - *C_i_* | -0.58 | < 0.0001 |
|  |  | *A_n_* - *g_s_* | 0.63 | < 0.0001 |
|  |  | *A_n_* - *E* | 0.64 | < 0.0001 |
|  |  | *A_n_* - ϕPSII | -0.01 | 0.91 |
|  |  | *A_n_* - *ψ* | -0.00 | 0.96 |
|  |  | *VPD_l_* - *g_s_* | -0.07 | 0.24 |
|  | rainy | *A_n_* - *T_leaf_* | 0.42 | < 0.0001 |
|  |  | *A_n_* - *VPD_l_* | 0.19 | 0.03 |
|  |  | *A_n_* - *PPFD_l_* | 0.81 | < 0.0001 |
|  |  | *A_n_* - *C_i_* | -0.65 | < 0.0001 |
|  |  | *A_n_* - *g_s_* | -0.17 | 0.12 |
|  |  | *A_n_* - *E* | -0.02 | 0.85 |
|  |  | *A_n_* - ϕPSII | -0.22 | 0.02 |
|  |  | *A_n_* - *ψ* | -0.39 | < 0.0001 |
|  |  | *VPD_l_* - *g_s_* | -0.54 | < 0.0001 |
| *E. grandiflora* | dry | *A_n_* - *T_leaf_* | 0.34 | <0.0001 |
|  |  | *A_n_* - *VPD_l_* | 0.31 | < 0.0001 |
|  |  | *A_n_* - *PPFD_l_* | 0.79 | <0.0001 |
|  |  | *A_n_* - *C_i_* | -0.81 | <0.0001 |
|  |  | *A_n_* - *g_s_* | 0.43 | <0.0001 |
|  |  | *A_n_* - *E* | 0.63 | <0.0001 |
|  |  | *A_n_* - ϕPSII | -0.33 | <0.0001 |
|  |  | *A_n_* - *ψ* | -0.14 | 0.02 |
|  |  | *VPD_l_* - *g_s_* | -0.19 | < 0.001 |
|  | rainy | *A_n_* - *T_leaf_* | 0.52 | < 0.0001 |
|  |  | *A_n_* - *VPD_l_* | 0.40 | < 0.0001 |
|  |  | *A_n_* - *PPFD_l_* | 0.90 | < 0.0001 |
|  |  | *A_n_* - *C_i_* | -0.88 | < 0.0001 |
|  |  | *A_n_* - *g_s_* | -0.43 | < 0.0001 |
|  |  | *A_n_* - *E* | 0.00 | 0.99 |
|  |  | *A_n_* - ϕPSII | -0.27 | <0.01 |
|  |  | *A_n_* - *ψ* | -0.32 | <0.001 |
|  |  | *VPD_l_* - *g_s_* | -0.47 | < 0.0001 |

**Supplementary Figure 1**. Relationship between WUE (*A_n_/E*) and *A_n_* and *E.* Response in the water use efficiency (*WUE*) to changes in net photosynthetic carbon gain (*A_n_*) and evapotranspiration (*E*) during the dry (grey) and rainy season (black) in *E. grandiflora* and *C. tessellata*. Relationship with *A_n_* during the dry season: r_s_ = 0.4*** and 0.6*** in *C. tessellata* and *E. grandiflora,* respectively; for the rainy season, r_s_ = 0.7*** in *C. tessellata* and *E. grandiflora*. Relationship with *E* during the dry season r_s_ = -0.2** and -0.1^N.S.^ in *C. tessellata* and *E. grandiflora,* respectively; for the rainy season, r_s_ = -0.5*** in *C. tessellata* and *E. grandiflora*. Significant differences are indicated: *, p < 0.05; **, p < 0.01; ***, p < 0.001; N.S. = not significant.

**Supplementary Figure 2**. Response in the net photosynthetic carbon gain (*A*) to changes in the stomatal conductance (*g*) and intercellular CO_2_ concentration (C_i_) during the dry (open symbols) and rainy season (closed symbols) in *E. grandiflora* and *C. tessellata*. Relationship with C_i_ during the dry season: r_s_ = -0.6*** and -0.7*** in *C. tessellata* and *E. grandiflora,* respectively; for the rainy season, r_s_ = -0.7*** and -0.8*** in *C. tessellata* and *E. grandiflora,* respectively. Relationship with *g* during the dry season r_s_ = 0.4*** and 0.5*** in *C. tessellata* and *E. grandiflora,* respectively; for the rainy season, r_s_ = -0.2* and -0.4* in *C. tessellata* and *E. grandiflora,* respectively (p < 0.05). Significant differences are indicated: *, p < 0.05; ***, p < 0.001.
